# Moral sensitivity of nursing students: a systematic review

**DOI:** 10.1186/s12912-024-01713-6

**Published:** 2024-02-06

**Authors:** Abdollah Zargar Shadi, Vanaki Zohreh, Mohammadi Eesa, Kazemnejad Anoshirvan

**Affiliations:** 1 Department of Nursing, Faculty of Medical Sciences, Tarbiat Modares University, Tehran, Iran; 2 Department of Nursing, Faculty of Medical Sciences, Tarbiat Modares University, Jalal Al Ahmad Ave, P.O.Box: 14115-111, Tehran, Iran; 3 Department of Biostatistics, Faculty of Medical Sciences, Tarbiat Modares University, Tehran, Iran

**Keywords:** Moral sensitivity, Ethical sensitivity, Nursing student, Rodgers’, Concept analysis, Education, Systematic review

## Abstract

**Background:**

Moral sensitivity is an essential criterion for nurses' professional competence, and it is effective in professional performance and the development of communication between nurse and patient. According to several definitions of moral sensitivity in nursing texts, each of them has examined moral sensitivity from a different aspect, and there are still uncertainties in the field of moral sensitivity of nursing students. Therefore, to clarify the characteristics and dimensions of the concept of moral sensitivity of nursing students, we used the method of concept analysis.

**Goal:**

To clarify and define moral sensitivity of nursing students.

**Method:**

For this systematic review, ten databases (PubMed, Scopus, Science Direct, Emerald, Springer, Proquest (open access), ERIC, SID, Irandoc, Magiran) were searched. They were published between 1990 and 2020. Inclusion criteria were having access to the full text of the article, types of studies (quantitative, qualitative, concept analysis, systematic review, and meta-analysis) related to the attributes, antecedents, and consequences of nursing students' moral sensitivity, medical students' moral sensitivity and Ethics education, articles being published in scientific journals in English or Persian language, texts published in non-peer-reviewed journals and letters to the editor were excluded from the data analysis. The methodological quality of the studies was assessed using the Mixed Methods Appraisal Tool 2018 and Prisma ScR Checklist 2020. Rodger's evolutionary concept analysis was used to conduct this study.

**Results:**

From 361 articles, 38 were included. The results showed moral sensitivity in nursing students improves through specialized ethical knowledge and presence in educational and professional settings. The attributes consist of honest and benevolent communication, compassionate professional practice, intuitive perceiving moral challenges, awareness of the responsibilities and moral consequences of decisions. The moral sensitivity of nursing students improves the quality of nursing care and the effective management of ethical challenges.

**Conclusion:**

Results can help develop nursing education theories and programs, design appropriate tools to evaluate this concept, and increase the quality of care and management of moral challenges in society and health systems.

**Limitation:**

This research has only examined Persian and English texts; also, accessing all the international databases was impossible, and more investigation in this field is required.

## Introduction

Issues such as the use of technology in care interventions, the increase in treatment costs and the number of patients, changes in the concepts of nursing and health needs [1], the increase in the number of older adults and end-of-life care [2], the patient's refusal to continue treatment are situations that constantly occur in the nursing profession; care providers must have high-quality performance as well as ethics. As the future workforce of the healthcare system [3], nursing students spend part of their training in hospitals and take care of patients alongside their nurses and instructors. Gastman (2002) believes that quality nursing care is influenced by moral performance and self-confidence, and the moral sensitivity of nurses should be improved to understand ethical issues and reach the highest level in order to respect people's values and rights [4]. In the clinical environment, in order to provide quality and holistic care, as well as to manage ethical challenges, students must have ethical sensitivity and prepare to deal with ethical challenges in their future roles [5, 6]. Ethical sensitivity makes the person providing care better deal with ethical issues and avoids unwanted and unethical results. Gastman (2002) believes that moral sensitivity is the ability to recognize the moral meaning of a specific situation and respond to it [4]. Weaver (2007) considers moral sensitivity to include behavioral concepts that refer to complex actions, intentions, emotions, and perceptions. In her study, she writes that moral sensitivity is a behavioral concept that has been described in the literature in different ways, such as caring response, skill in identifying the moral dimension of caring, intuition about the comfort and well-being of others, and the component of moral care [7]. Lovett and Jordan(2010) stated that moral sensitivity is "the ability to recognize the existence of moral issues in real-world situations [8]." Park et al. (2012) believe that moral sensitivity is caring for the well-being of others and is more than a cognitive ability [9]. Shayeste Fard et al. (2020) believe that this feature is a cognitive-critical process [10].

Now, according to the definitions of the concept of moral sensitivity in nurses, the characteristics of this concept in nurses and nursing students are somewhat the same due to the common work field. It is also essential to pay attention to the fact that, in addition to interacting with the patient, nursing students are also interacting with the professor, classmates, and treatment staff. As a result, the moral context of his interaction with nurses is different, which affects the moral sensitivity characteristics of nursing students. Considering the importance of moral sensitivity in professional practice and in order to develop nursing ethics, a set of structures and concepts related to it should be designed and perfected. A precise definition of the concept of moral sensitivity and increasing awareness in the field of this concept help to interpret it more appropriately. The use of concept analysis can lead to transparency and give meaning to the concept of moral sensitivity of students. This clarity and transparency and increasing understanding and updating knowledge can create a scientific basis for practice and evidence-based interventions, develop theories and educational programs and design appropriate tools for evaluating this concept, examine the level of moral sensitivity of nursing students, and finally increase quality of care and management of ethical challenges in society and health systems. According to the stated contents, this research was conducted with the aim of analyzing the concept of moral sensitivity of nursing students.

## Method

Concept analysis in nursing is used to clarify concepts in theory, practice, and research and to reach precise theoretical and operational definitions for research [11]. Various approaches to concept analysis have been described, for example, Rogers (1989), Chin and Kramer (1991), and Walker and Avant (1995). In this study, Rogers' concept analysis method was used because Rogers believes that concepts are dynamic and change and develop over time based on context [12]. Weaver (2008) believes that Rogers' method of concept analysis is called "evolutionary" and focuses on the idea that concepts evolve in a specific context, which may be disciplinary, cultural, or theoretical, and this development continues [13]. The concept of moral sensitivity in students is a dynamic concept that may change based on time and place, professional guidelines, and social rules. The concept's attributes, antecedents, and consequences are clearly defined in Rogers' method.

### The stages of Rodgers’ evolutionary concept analysis include the following steps:

1. Determining the desired concept and identifying surrogate and related terms
2. Determining and selecting the appropriate scope and time for data collection
3. Data collection to achieve the attributes of the concepts, antecedents, consequences, and underlying foundations of the concept
4. Data analysis based on the above characteristics
5. Expression of appropriate example about the concept
6. Provide hypotheses for further evolution of the concept [14].

### Determine the desired concept and identify surrogate and related terms

Considering that moral sensitivity is significant in the performance of nursing students who are the future builders of this profession and is one of the basic concerns in nursing education and clinical care, moral sensitivity of nursing student chose as the desired concept. In this study, nursing students are students studying or about to graduate. The texts were reviewed to determine the surrogate and related terms. It can be said that surrogate words express the idea of ​​a concept through words other than the concept that the researcher considered in his study [12]. In the texts, moral sensitivity and ethical sensitivity are used interchangeably [15].

Regarding related words, they share the same concept but do not have the same characteristics [12]. Related concepts include part of the relationships and dependencies of the main concept, they are close to the main concept, but they alone do not have all the features of the studied concept [16]. In the review of the texts, it was determined that the words "moral judgment," "moral motivation," "moral character," "moral decision-making," and "moral reasoning" are related concepts; this means that they are common in terms of moral concepts, but describe different characteristics. To prove this, we can say, for example, Rest and his colleagues state in their study that ethical decision-making consists of four components: (1) Moral sensitivity; (2) Moral judgment; (3) Moral motivation; (4) Moral character [15]. It means that moral sensitivity is related to three other words in the field of ethics. In another study, it is stated that moral sensitivity and moral motivation are related [17]. Moral sensitivity is one of the critical concepts in theories of moral development and is likewise associated with moral reasoning and moral judgment [18]. In this regard, by searching the texts, surrogate and related concepts of moral sensitivity of nursing students were identified. Articles related to surrogate concepts were included in the final analysis, and retrieved documents about related concepts were excluded from the final analysis. After completing the above steps, the research team checked and approved the findings.

### Determining and selecting the appropriate scope and time for data collection

The second step in Rogers' approach is to determine the appropriate field and time for data collection; in the present study, the research team chose nursing as the desired discipline and compared the results of its articles with the medical discipline and ethics education. The reason for choosing the discipline of medicine and ethics education is because, according to the literature, the concept of moral sensitivity in the three domains of nursing, medicine, and ethics education are closely related.

### Appropriate time

Rest and his colleagues expressed the concept of moral sensitivity based on Kohlberg's theory in 1986. In 1990, they published an article about medical students' moral sensitivity; in 1995, they published their article titled "Professional Ethical Progress" [19]. Therefore, the years 1990–2020 were considered for the literature review in this study.

### Data collection to achieve the attributes of the concepts, antecedents, consequences, and underlying foundations of the concept

Since access to all international databases was impossible, the following domestic and international databases were used to collect data: PubMed, Scopus, Science Direct, Emerald, Springer, ProQuest (open access), ERIC, SID, Irandoc, and Magiran.

### Search strategy

The keywords were “Moral”, “Ethical”, “sensitivity”, “nursing student”, “student nurse”, “medical student”, “ethics education”, “ethical issue”, “nursing practice” which were searched individually, and in combination with AND/OR.

Moral OR ethical AND sensitivity AND nursing student OR student nurse AND medical student AND ethics education AND ethical issue AND nursing practice.

### Study selection

After downloading the articles, first, the titles and abstracts of the articles match the inclusion criteria. Two authors independently reviewed the full text of the articles and discussed discrepancies until agreement was reached. Study details were extracted from articles and charted in a table, which was used to decide on study inclusion.

### Inclusion/exclusion criteria:

**Table:** 

**Inclusion criteria**	**Exclusion criteria**
1- Having access to the full text of the article 2-Types of studies (quantitative, qualitative, concept analysis, systematic review, and meta-analysis) related to the attributes, antecedents, and consequences of nursing students' moral sensitivity, medical students' moral sensitivity, and Ethics Education 3- Articles being published in scientific journals in the period 1990–2020 4- Articles being published in English or Persian language	1-Texts published in non-peer-reviewed journals and letters to the editor were excluded from the data analysis

### Search result

**Fig. 1:**
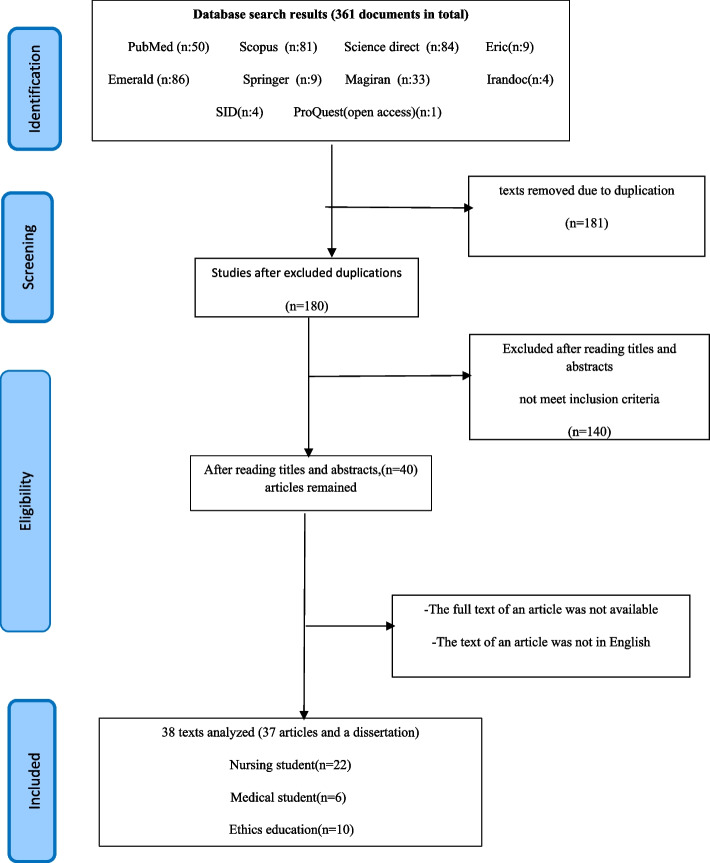
Flow chart of text screening process

At this stage, 361 articles were listed: 52 related to education, 83 related to medicine, and the rest were nurses. Of these, 181 duplicate articles between databases were excluded. Another researcher reviewed the remaining articles simultaneously and separately. Next, after reviewing the titles, abstracts, and text of the articles, we found that, among the remaining papers, 140 did not mention the moral sensitivity of nursing and medical students and ethics education. Finally, 40 articles remained. In the next step, despite the checks carried out by the university library, the full text of one of the articles was unavailable, and the text of another article was not in English, so we analyzed 38 documents (37 articles and one thesis). Out of 38 articles, six were related to medical discipline, ten were related to ethics education, and 22 were related to nursing discipline. The methodological quality of the studies was assessed using the Mixed Methods Appraisal Tool (MMAT) [20] and PRISMA-ScR [21]. Of these documents, nine articles were in Persian, and the rest were in English (Table 1 and Fig. 1).

**Table 1:** Search results in different databases

Row	Bases	Non-relevant articles	Duplicate articles	Relevant articles	Thesis	Non-access	Non-English languages
1	PubMed	16	33	1			
2	Scopus	22	31	26		1	1
3	Science direct	38	45	1			
4	Emerald	34	52	-			
5	Eric	4	5	-			
6	Springer	4	5	-			
7	Magiran	17	7	9			
8	Irandoc	4	-	-			
9	SID	1	3	-			
10	ProQuest(open access)	-	-	-	1		
	Total	140	181	37	1	1	1

### Quality assessment process

The methodological quality of the included studies was assessed independently by two authors using the Mixed Methods Appraisal Tool (MMAT) [20] and PRISMA-ScR [21]. The MMAT was designed to assess various empirical studies in five categories, including qualitative studies, randomized controlled trials, non-randomized studies, quantitative, descriptive studies, and mixed methods studies. This instrument consists of 5 items for each category, each of which could be marked as yes, no, or not known. The scoring system provides that the “yes” answer is scored as 1, and all other answers are scored as 0. A higher score indicates higher quality. When evaluating the final scores in terms of quality, scores above half (more than 50%) were considered high quality. The PRISMA-ScR checklist contains 20 essential reporting items and 2 optional items to include when completing a scoping review. Scoping reviews serve to synthesize evidence and assess the scope of literature on a topic. Among other objectives, scoping reviews help determine whether a systematic review of the literature is warranted. Liberati and colleagues (2009) noted that PRISMA focuses on the methods that authors can utilize to ensure the transparent and complete reporting of systematic reviews [22]. Finally, the data were analyzed by extracting the textual content of the articles in the context of the study.

## Data analysis

Data review and extraction questions: How is the moral sensitivity of nursing students defined? What are the attributes, antecedents, and consequences of nursing students' moral sensitivity?

Since analysis means examining a whole within its parts and elements to be better understood, its default is that the concepts exist in the texts. There are explanations and definitions, but the analysis is to improve it to a higher level.

After selecting relevant articles and sorting them according to the year of publication, the text of each article was read several times by the research team. In selecting and sorting the articles, all the papers were carefully registered and classified, and we recorded the reason for separating the pieces next to them. The themes that did not meet the inclusion criteria were removed from the collection to avoid making a mistake in this field. The research team supervised all the stages of the analysis. We analyzed the text of the related articles based on the thematic analysis method. [22]. Meaning units were identified based on their relevance to the attributes, antecedents, and consequences of moral sensitivity among nursing students and extracted related codes. Related concepts were excluded from this process. The main codes were obtained by combining similar primary codes, and the subcategories were obtained by combining similar main codes. The subcategories were reviewed, and similar ones were merged after repeated comparisons to form the main categories (Table 2).

**Table 2:** An example of data analysis of an ability to perceive intuitively moral challenges

Meaning unit	Code	Subcategory	Category
Moral sensitivity is the ability to recognize the presence of moral issues in real-world situations [23]	An ability to recognize moral issues in real-world situations	An ability to recognize moral challenges	Ability to perceive moral challenges intuitively
Moral sensitivity, which is defined as the person’s ability to identify moral conflicts and sensory and perceptual perceptions of individuals in vulnerable situations [24]	The person’s ability to identify moral conflicts, and intuitive perceptions of individuals in vulnerable situations	Intuitive perceptions of individuals in vulnerable situations	

Therefore, 238 primary codes were extracted; similar codes were removed, and 77 main codes remained. Finally, 14 subcategories and 9 main categories (2 for antecedent, 2 for consequence, and 5 for attributes) were extracted.

## Results

Attributes of the concept of moral sensitivity among nursing students.

Attributes form a deep insight into the concept [25]. Regarding moral sensitivity among nursing students, five main attributes and eight sub-attributes were obtained from data analysis.

(Table 3).

**Table 3:** The main attributes and sub-attributes of the concept of moral sensitivity among nursing students based on data analysis

Main attributes	Sub-attributes
Ability to perceive moral challenges intuitively	Ability to recognize moral challenges, Intuitive perception of vulnerable situations
Compassionate professional practice	Behaving compassionately with people, Having professional behavior
Awareness of responsibilities and moral consequences of decisions	Recognizing responsibilities in moral situations, Being aware of the moral consequences of decisions
Honest and benevolent interpersonal communication	Ability to communicate interpersonally, Honest and benevolent behavior
An ability to think critically about moral-legal challenges	Thinking about ethical issues of care, Critical attention to legal-ethical issues

### Explanation of the ability to perceive moral challenges intuitively

A nursing student who can recognize moral challenges knows moral values correctly and recognizes ethical dilemmas in professional performance and learning environment quickly without intermediaries self-consciously. This attribute has two sub-attributes: the ability to recognize moral challenges and the intuitive perception of individuals in vulnerable situations.

Ability to recognize moral challenges: A morally sensitive nursing student can detect ethical violations, positively affecting a moral environment [6]. Moral Sensitivity is a security alert to identify ethical issues and an ability to understand the conflict between individuals' beliefs, perceptions, interests, and well-being [26]. A morally sensitive student can identify value systems [6]. Ethical sensitivity is the awareness of aspects of any moral situation [27] and the ability to frame ethical issues [28].

Intuitive perception of individuals in vulnerable situations: A morally sensitive nursing student has a caring behavior and recognizes the ethical dimension of care, perceiving the comfort and well-being of others intuitively [26]. Moral sensitivity is a personal and intuitive competence [29] and a contextual, intuitive, and immediate perception of the patient in a vulnerable situation [30].

### Explanation of compassionate professional practice

A nursing student who has a compassionate professional practice is that he behaves with respect and support, kindness, positive attention, and unconditional love towards himself and others. This attribute has two sub-attributes: behaving compassionately with people and having professional behavior.

#### Behaving compassionately with people

A morally sensitive student focuses on the emotional skills required for awareness of moral problems [31] and pays attention to the patient. Moral sensitivity is dependent on the personal characteristics and beliefs of people. Kindness, honesty, benevolence, and help to others cause the student to set goals and behave morally with patients, their families, and colleagues [32].

#### Having professional behavior

Park et al. (2012) believe that moral sensitivity is caring for the well-being of others, and it is more than cognitive ability [9]. It is a behavioral concept involving complex actions, intentions, emotions, and perceptions [32].

### Explaining awareness of responsibilities and ethical consequences of decisions

A morally sensitive nursing student knows the ethical responsibilities and consequences of decisions. They are responsible for and direct their practices, take steps toward ethical and legal standards, and are aware of the moral consequences of their decisions. This attribute has two sub-attributes: recognizing responsibilities in the moral situation and being aware of the moral consequences of decisions.

#### Recognizing responsibilities in moral situations

A morally sensitive student is aware of roles and responsibilities in moral situations [33], observes professional ethics while performing responsibility [3], and has self-awareness of responsibilities in moral situations [28].

#### Being aware of the moral consequences of decisions

A morally sensitive student is mindful of how their behavior affects the comfort and well-being of individuals [28]. These students have insights into the moral consequences of decisions and actions [24]. These students can evaluate the responses and feelings of others and are aware of their potential behaviors [30].

### Explanation of honest and benevolent interpersonal communication

A morally sensitive nursing student can communicate with people honestly and benevolently. This attribute has two sub-attributes: the ability to communicate interpersonally and honest and benevolent behavior.

#### Ability to communicate interpersonally

A morally sensitive student knows how to communicate with patients [34] and other students [30]. Communication is an essential part of moral sensitivity [24]. A morally sensitive student communicates with patients [35], cooperates sincerely with them [10], and observes and pays attention to interpersonal communication [36].

#### Honest and benevolent behavior

Morally sensitive students have honest and benevolent behaviors [37]. They observe justice in their behavior [38]. They also behave beneficently [28].

### Explanation of ability to think critically about ethical-legal challenges

A morally sensitive student can think critically about ethical-legal challenges. This student can ask appropriate questions about ethical challenges and gather relevant information. Then, they categorize them creatively, reason logically, and come to a reliable conclusion. This attribute has two sub-attributes of thinking about ethical issues of care and critical attention to legal-ethical issues.

#### Thinking about ethical issues of care

A morally sensitive student thinks about ethical and legal issues [27]. Moral sensitivity includes thinking, reflection, honesty, judgment during care conflict, and decision [38].

#### Critical attention to legal-ethical issues

A morally sensitive student discusses ethical-legal issues with others [27] and cares about ethical issues [39]. According to Shayesteh Fard et al. (2020), this is a cognitive-critical process [10].

### Antecedents of the concept of moral sensitivity among nursing students

Antecedents occur before the occurrence of the concept. According to literature analysis, antecedents of the concept of moral sensitivity among nursing students include specialized-ethical knowledge and presence in professional-educational settings (Table 4).

**Table 4:** Antecedents of the concept of moral sensitivity of nursing students based on data analysis

-Having specialized-ethical knowledge
-Presence in professional-educational settings

#### Explanation of having specialized-ethical knowledge

Students’ knowledge and awareness of moral sensitivity should be increased with appropriate training. Studies addressed the effect of training on moral sensitivity and reported that lack of training could be an obstacle to developing moral sensitivity and professional ethics.

Awareness of professional knowledge, existing laws, and ethical situations can lead to ethical sensitivity [27]. Knowing the moral dimensions of a situation and distinguishing it from another problem requires moral knowledge [40]. Professional [39] and moral knowledge [6] are necessary to acquire moral sensitivity.

Explanation of presence in professional-educational settings: Nursing students can develop their moral sensitivity by being present in the educational environment [41]. Presence in the professional and clinical environment [39] paves the way for developing the concept of moral sensitivity among nursing students. According to Karimi et al. (2016), a clinical setting is effective in moral sensitivity [41].

### Consequences of the concept of moral sensitivity among nursing students

Consequences occur as a result of the occurrence of the concept [16]. Consequences of the concept of moral sensitivity among nursing students are the promotion of quality of nursing care and effective management of moral challenges (Table 5).

**Table 5:** Consequences of the concept of moral sensitivity among nursing students based on data analysis

Main category	Subcategory
Promotion of the quality of nursing care	Improvement of quality of nursing care
	An ability to identify patient needs
Effective management of moral challenges	Management of moral care problems
	Moral development

#### Explanation of the promotion of the quality of nursing care

One of the consequences of the concept of moral sensitivity among nursing students is the promotion of the quality of nursing care. This consequence has two subcategories: improvement of the quality of nursing care and an ability to identify patient needs.

#### Explanation of improvement of the quality of nursing care

Ethical sensitivity, the foundation of ethics in nursing, provides the basis for effective and ethical care. Ethical sensitivity skills training increases students' moral sensitivity, improving the quality of nursing care [24] and moral practice [32]. Therefore, nursing students provide moral care to patients [42] and meet their needs morally [43]. As a result, moral sensitivity increases the commitment to a patient [32]and maintains their safety [43].

#### Explanation of the ability to identify the patient's needs

Morally sensitive nursing students are aware of the patient's verbal and non-verbal behaviors [36] and focus on the patient's needs to meet them [33]. This consequence leads to better cognition of the patient [32].

The second consequence is the effective management of moral challenges. This consequence has two subcategories of management of moral care problems and ethical development.

#### Explanation of management of moral care problems

Escolar et al. (2018) found that moral sensitivity increased nursing students’ abilities and skills in effectively managing moral distress [23]. They also could solve problems and prevent conflicts [43]. Nursing students’ ethical sensitivity leads to the management of ethical dilemmas and the solution of problems related to patient care [23].

Explanation of ethical development: Ethical sensitivity of nursing students leads to moral maturity [29] and reduces moral distress [23].

## Discussion

The purpose of this study is to provide a clear and comprehensive definition of the concept of moral sensitivity in nursing students. According to the obtained results, moral sensitivity is a concept beyond the ability to recognize moral challenges and be sensitive to moral issues. The antecedents of this concept are specialized-ethical knowledge and presence in the environment. Educational-professional activities are the basis for the emergence of honest and benevolent communication characteristics, compassionate professional performance, the ability to intuitively know ethical challenges, awareness of responsibilities and ethical consequences of decisions made, and the ability to think critically about ethical-legal challenges in nursing students. The emergence of such characteristics leads to the improvement of the quality of nursing care and the ability to manage ethical challenges effectively.

In their study, Shayeste Fard et al. (2020) described the characteristics of moral sensitivity of nursing students as moral perception, effectiveness, critical cognitive process, and sincere cooperation. In explaining the characteristics of sincere cooperation, they stated that the meaning of cooperation is sincere with his peers and clinical instructor to face moral challenges. They share their feelings and lack of skills and knowledge with their classmates when dealing with moral challenges. Students can understand the moral nature of the situation and respond to it through the inevitable informational and emotional support of their instructor. They considered the moral sensitivity of nursing students to be the dynamic ability to make moral decisions in ambiguous situations in the presence of their peers and instructors [10]. According to the results, it can be said since the number of qualitative studies that are the result of students' own lived experiences in the field of students' moral sensitivity is small, the results of these studies did not influence the analysis process to specify the concept of moral sensitivity. From the comparison of the results of the studies, some characteristics, such as moral understanding or critical cognitive processes, in line with the results of the present study.

In another study, Muramatsu et al. (2019) stated the characteristics of nursing students' moral sensitivity as "respect for people", "distributive justice" and "maintaining patients' confidentiality" [38]. Borhani and his colleagues (2013) stated guidance and inner support, recognition, correct reaction, responsiveness, and interest as the characteristics of moral sensitivity of nursing students [32]. From the comparison of the results of studies, the characteristics of moral sensitivity of nursing students in our study is more comprehensively expressed. In the present study, honest and benevolent communication, compassionate professional practice, intuitive perceiving moral challenges, awareness of the responsibilities and moral consequences of decisions, and the ability to think critically about moral-legal challenges have been mentioned, which provide added value for clarifying the concept of ethical sensitivity of nursing students.

According to the obtained results, it was determined that there is no difference between the moral sensitivity of nursing students and nurses based on the analysis of the texts; at the same time, it seemed that, because nursing students interact with the patient, the professor, classmate, treatment staff so the moral context of his interactions with nurse and characteristics of their moral sensitivity is also different. Among the reasons for this result, it can be stated that the authors of the analyzed articles probably had a common view of the moral sensitivity of nurses and nursing students. On the other hand, the text of the articles is mainly adapted from sources of ethics and other disciplines, which can lead to the creation of common knowledge, which is related to the weakness of our professional texts. This issue shows the necessity of conducting more qualitative studies and benefiting from the lived experiences of nursing students.

### Application of results in management

Based on the findings of the study (antecedent: presence in educational-professional environments and having specialized and ethical knowledge), nursing managers can, with appropriate planning and removing oppressive factors, provide the context for students and nurses to attend appropriate educational and professional environments and acquire the necessary specialized and ethical knowledge to achieve and develop the specified characteristics.

### Application of results in nursing practice

Since nursing students are in close contact with the medical staff in clinical settings and a student with moral sensitivity can think critically about ethical challenges, the medical staff must be precise and sensitive to the observance of moral values so that the students do not have problems and conflicts and students do not criticize them. In this way, moral sensitivity is cultivated in students and medical staff.

### Application of results in research

Considering the determination and definition of dimensions and characteristics of moral sensitivity, it is suggested to design the tool after a qualitative field study to clarify practical and objective examples.

### Limitations

This research has only examined Persian and English texts. Therefore, some essential texts in other languages needed to be included; also, accessing all the international databases was impossible, and more investigation in this field is required.

### Comparing moral sensitivity in different professional contexts

The concept of moral sensitivity in the texts related to nursing students, medical students, and ethics education was analyzed and compared. The results showed that most medical texts focused on the ability to recognize moral issues in clinical cases and determine the importance of challenges or the ability to identify the challenges in medical ethics. Ethics education texts focused on the importance of moral education, the type of teaching method, and the use of hybrid teaching methods as influential factors in developing moral sensitivity. However, nursing literature focused on the ability to perceive moral challenges intuitively, compassionate professional practice, honest and benevolent communication, awareness of ethical responsibilities and consequences of decisions, and the ability to think critically about moral-legal challenges.

### Changes the concept of nursing students' moral sensitivity over time

Moral sensitivity has gone through three periods of evolution. In the range of 1990–1999, the results of the analysis of the concept of moral sensitivity show that most of the texts referred to the cognitive dimension of the concept of moral sensitivity, such as the ability to recognize ethical challenges [19] and the ability to recognize the moral aspects of situations [44]. In the range of 2000–2009, ethical texts about moral sensitivity point to the issue that in defining this concept, in addition to understanding and recognizing ethical issues, the emotional dimension is also important. In the emotional aspect of moral sensitivity, interest and attention to the patient is an important issue. This concept and other cognitive and skill dimensions concepts propose as the constituent element of moral sensitivity [7]. To be aware of the ethical challenge, it is necessary to focus on emotional skills [31]and the ability to make intelligent and compassionate decisions during caregiving [7]. In the range of 2010 to 2020, the texts also mentioned another issue about the characteristic of moral sensitivity, that moral sensitivity, in addition to cognitive and emotional factors, is a cognitive-critical process [10], thinking and reflection, honesty, judgment at the time of care conflict, decision making [38], discussion and negotiation about moral and legal issues [27].

### An appropriate example of moral sensitivity among nursing students (The researcher designed this model based on the moral sensitivity attributes, antecedents, and consequences of nursing students obtained in this study)

One day, the student went to the patient's bedside with his instructor to give medicine to the patient. When the patient saw the drug that had changed color, he was worried that he was being given the wrong medication and asked. The student calmly and kindly explained that this is the same medicine as before because his company is different, the color has changed, and there is nothing to worry about (attributes: professional performance with compassion and kindness). The patient was discharged and needed a wavy mattress at home. During the discharge training, the student told him he should have a wavy mattress at home. Still, he realized the patient and his companion had problems getting a corrugated mattress. After that, the student followed up on where the patient's companion could rent this device, which he introduced to them (attributes: awareness of responsibility). One day, while entering the ward with his nursing students, the instructor realized that the patient had low-grade epilepsy. He asked his students to raise the railing next to the bed immediately. He explained to the students the possibility of convulsions, falling from the bed, and paying attention to the patient's safety (antecedent: having specialized and ethical knowledge, being in educational-professional environments). The patient had come from a distant city for an angiography and was very tired. He requested the staff to perform the operation before the others. One of the nurses explained to him that the work was done in turn. The patient started screaming. The nursing student was very upset about the patient's condition and went to talk to him and explained that all the angiography appointments in the morning would be completed by 11/30. Their work will be done early, and he tried to calm the patient and make everyone work calmly that day (consequence: the ability to manage the moral challenge effectively).

### Provide hypotheses for further evolution of the concept

Finally, it can be said that moral sensitivity in nursing students is a multidimensional concept including cognitive, emotional and behavioral dimensions that requires specialized-ethical knowledge and presence in educational-professional environments to establish honest and benevolent communication, provide professional and compassionate practice, perceive ethical challenges intuitively, be aware of ethical responsibilities and consequences of decisions, and think critically about ethical-legal challenges. These attributes will improve the quality of nursing care and effectively manage ethical challenges.

## Conclusion

This study analyzed and clarified the perceptions of moral sensitivity among nursing students and presented the attributes, antecedents, and consequences. Concept analysis of the moral sensitivity of nursing students indicates that specialized-ethical knowledge and presence in educational-professional environments lead to moral sensitivity among nursing students. This concept has attributes such as the ability to perceive moral challenges intuitively, compassionate professional practice, the ability to think critically about legal and ethical challenges, awareness of the ethical responsibilities and consequences of decisions, and interpersonal honest, benevolent communication. In addition, the moral sensitivity of nursing students improves the quality of nursing care and the effective management of ethical challenges. The study results can help develop nursing education theories and programs, design appropriate tools to evaluate this concept and increase the quality of care and management of moral challenges in society and health systems.

## Data Availability

The datasets generated and/or analysed during the current study are not publicly available due privacy and ethical restrictions, but are available from the corresponding author on reasonable request.

## References

[1] Borhani F, Keshtgar M, Abbaszadeh A (2015). Moral self-concept and moral sensitivity in Iranian nurses. J Med Ethics Hist Med.

[2] Robichaux C (2012). Developing ethical skills: from sensitivity to action. J Critical Care Nurse.

[3] Yeom H-A, Ahn S-H, Kim S-J (2017). Effects of ethics education on moral sensitivity of nursing students. Nurs Ethics.

[4] Gastmans C (2002). A fundamental ethical approach to nursing: some proposals for ethics education. Nurs Ethics.

[5] Cannaerts N, Gastmans C, Casterlé BDd (2014). Contribution of ethics education to the ethical competence of nursing students: Educators’ and students’ perceptions. J Nursing ethics..

[6] Baykara ZG, Demir SG, Yaman S (2015). The effect of ethics training on students recognizing ethical violations and developing moral sensitivity. J Nurs Ethics.

[7] Weaver K (2007). Ethical sensitivity: state of knowledge and needs for further research. J Nursing ethics.

[8] Lovett BJ, Jordan AH (2010). Levels of moralisation: A new conception of moral sensitivity. J Moral Educ.

[9] Park M, Kjervik D, Crandell J, Oermann MH (2012). The relationship of ethics education to moral sensitivity and moral reasoning skills of nursing students. Nurs Ethics.

[10] Shayestehfard M, Torabizadeh C, Gholamzadeh S, Ebadi A. Ethical sensitivity in nursing students: Developing a context–based education. Elect J Gen Med. 2020;17(2):1–12 (persian).

[11] Fitzpatrick JJ, Wallace M (2006). Encyclopedia of nursing research: Springer Publishing Company.

[12] Rogers B. Concept analysis: An evolutionary view In Rogers B & Knafl KA (2nd Eds.), Concept development in nursing: Foundations, techniques, and application (pp. 77–102). Philadelphia: WB Saunders Co.[Google Scholar]; 2000.

[13] Weaver K, Mitcham C (2008). Nursing concept analysis in North America: state of the art. Nurs Philos.

[14] Rodgers BL, Knafl KA (1999). Concept development in nursing: Foundations, techniques, and applications: WB Saunders Co.

[15] Lützén K, Dahlqvist V, Eriksson S, Norberg A (2006). Developing the concept of moral sensitivity in health care practice. J Nursing ethics.

[16] Tofthagen R, Fagerstrøm LM (2010). Rodgers’ evolutionary concept analysis–a valid method for developing knowledge in nursing science. Scand J Caring Sci.

[17] Tymieniecka A-T (1986). The moral sense and the human person within the fabric of communal life. The Moral Sense in the Communal Significance of Life: Springer.

[18] Pedersen LJT (2009). See no evil: moral sensitivity in the formulation of business problems. Bus Ethics Eur Rev.

[19] Hébert P, Meslin EM, Dunn EV, Byrne N, Reid SR (1990). Evaluating ethical sensitivity in medical students: using vignettes as an instrument. J Med Ethics.

[20] Hong QN, Fàbregues S, Bartlett G, Boardman F, Cargo M, Dagenais P (2018). The Mixed Methods Appraisal Tool (MMAT) version 2018 for information professionals and researchers. Educ Inf.

[21] Tricco AC, Lillie E, Zarin W, O'Brien KK, Colquhoun H, Levac D (2018). PRISMA extension for scoping reviews (PRISMA-ScR): checklist and explanation. Ann Intern Med.

[22] Vaismoradi M, Turunen H, Bondas T (2013). Content analysis and thematic analysis: Implications for conducting a qualitative descriptive study. Nurs Health Sci.

[23] Escolar-Chua RL (2018). Moral sensitivity, moral distress, and moral courage among baccalaureate Filipino nursing students. Nurs Ethics.

[24] Jalili F, Saeidnejad Z, Aghajani M (2020). Effects of spirituality training on the moral sensitivity of nursing students: A clinical randomized controlled trial. Clinical Ethics.

[25] Walker LO, Avant KC (2005). Strategies for theory construction in nursing: Pearson/Prentice Hall Upper Saddle River NJ.

[26] Chocolate CP (2013). The effect of ethics education on the development of ethical sensitivity of undergraduate accounting students: an exploratory study in angola: Argosy University/Sarasota.

[27] Borhani F, Mohsenpour M (2011). Barrier to acquiring ethical sensitivity: perceptions of nursing students. J Medical Ethics Journal..

[28] Spekkink A, Jacobs G. The development of moral sensitivity of nursing students: A scoping review. Nurs Ethics. 2020;28(5):1–18. 10.1177/0969733020972450 33325340

[29] Tuvesson H, Lützén K (2017). Demographic factors associated with moral sensitivity among nursing students. Nurs Ethics.

[30] Comrie RW (2012). An analysis of undergraduate and graduate student nurses’ moral sensitivity. J Nursing ethics.

[31] Morton KR, Worthley JS, Testerman JK, Mahoney ML (2006). Defining features of moral sensitivity and moral motivation: Pathways to moral reasoning in medical students. J Moral Educ.

[32] Borhani F, Abbaszadeh A, Mohsenpour M (2013). Explaining the Meaning of Ethical Sensitivity in Nursing Students: A Qualitative Research. J Med Ethics.

[33] Lee HL, Huang S-H, Huang C-M (2017). Evaluating the effect of three teaching strategies on student nurses’ moral sensitivity. Nurs Ethics.

[34] AbbasZadeh A, Borhani F, Moazen NL (2010). The comparison of the level of moral sensitivity in nursing students and nursing staffs in Kerman in 1389.

[35] Kim W-J, Park J-H (2019). The effects of debate-based ethics education on the moral sensitivity and judgment of nursing students: A quasi-experimental study. Nurse Educ Today.

[36] Jasemi M, Rasoulgoli REZ, Khalkhali H (2020). Effects of Teaching Nursing Codes of Ethics through Lecture on Moral Sensitivity and Moral Performance of Nursing Students–A single blind, Quasi Experimental Study. Pak J Med Health Sci..

[37] Kohansal Z, Avaznejad N, Bagherian B, Jahanpour F (2018). Evaluation of moral sensitivity in nursing students of Bushehr University of Medical Sciences in 2016. Iran J Med Ethics Hist Med.

[38] Muramatsu T, Nakamura M, Okada E, Katayama H, Ojima T (2019). The development and validation of the Ethical Sensitivity Questionnaire for Nursing Students. J BMC Med Educ.

[39] Mostafavian Z, Gholampour J, Faraj Pour A, Akbari Farmad S, Rahchamani MA (2019). Comparison of Moral Sensitivity among last year Nursing Students and Nurses Working at teaching Hospitals of Islamic Azad University of Mashhad. Educ Ethics Nurs.

[40] Ersoy N, Gündoğmuş ÜN (2003). A study of the ethical sensitivity of physicians in Turkey. J Nurs Ethics.

[41] Karimi NM, Tavakkoli N, Borhani F, Mohsenpour M (2016). Ethical sensitivity: A comparison between the nursing students and nurses of Azad University.

[42] Mousavi S, Mohsenpour M, Borhani F, Ebadi M (2015). Ethical Sensitivity of nurses and nursing students working in Aja University of Medical Sciences.

[43] Gülnar E, Özveren H, Özden D (2020). The relationship between moral sensitivity and medical errors attitude in nursing students. J Forensic Leg Med.

[44] Oddi LF, Cassidy VR, Fisher C (1995). Nurses' sensitivity to the ethical aspects of clinical practice. J Nursing Ethics.

